# Preparation of the Mouth for Artificial Teeth

**Published:** 1856-10

**Authors:** H. R. Smith


### ARTICLE XI.

*Preparation of the Mouth for Artificial Teeth.* By H. R. Smith, M. D., D. D. S.

Dear Doctor:

There is a subject which appears to me to be of great importance, on which very little is said through our public journals. Important, because one of our most common operations is dependent upon it; and one which if not properly understood, will bring failures if not disastrous results.

It is a subject which even some of the older members of our profession pass by without due regard, and one which costs the tyro many years of practice, and many failures, to duly appreciate.

The subject to which I allude is the preparation of the mouth for artificial teeth, and the care to be observed after they are inserted. Too often do we find work failing and being thrown aside, not from any defect in the mechanical piece, but from the remaining natural teeth giving away, or gums receding from the artificial fixture. To such an extent has this been the case, that even some of our leading dentists are "down" on the mechanical dentist, and abuse not only the work but the operator.

I think this may all be referred to the fact of neglect in laying the foundation properly for the work. Of the importance of saving the natural organs, I will not speak, for all are, or should be aware that, such should be our greatest aim. But with all of our good operations, and salutary advice, many teeth will fail and substitutes will be called for.

The question then comes up, how shall we perform the operation to be of the greatest benefit to our patient? Here will come the test between the mere mechanic and the skillful operator.

It has been asserted by the "anti-artificial" dentist that a jeweler, or any other good worker in metals, could make a dental substitute. Such may be the case so far as outward appearances are concerned, but the "goodness" of an operation does not depend on that alone, but on the permanent value to the patient. And to insure that result, more than a knowledge of mechanics is necessary.

First, an intimate knowledge of chemistry is necessary, in order to understand the chemical action constantly going on in the mouth, which, by changing the fluids and particles of food, may destroy the natural teeth and membranes. And secondly, what action is produced when a metal is brought into connection with the teeth and these fluids.

A knowledge of anatomy and physiology is necessary in order to know the form of parts involved and the changes which take place from time to time in the alveola and surrounding parts; and of pathology and therapeutics, to understand what the changes are which are produced by disease, and what remedial agents may be brought to bear to restore the parts to health.

When these subjects are fully understood, then, and then only, can the good mechanic succeed in making his work good and useful.

Oftentimes do we have patients present themselves to have aching teeth and roots removed, diseases of the mouth and gums cured, teeth plugged and artificial teeth inserted, and all in the space of a few days or weeks, and too often does the "mighty dollar" tempt the operator to undertake the job. The work is done and the money is secured, and the conscience of the operator (if he has one) is quieted by the reflection that the work looked well and "paid."

Should it be any wonder that such operations fail? If they do not, it will not be from any science or skill of the operator, but from the power of nature to overcome difficulties, and more or less accommodate herself to circumstances.

It is a practice amongst very many of our first operators to insert what are termed temporary sets of artificial teeth, almost immediately after the teeth have been extracted. It is a practice which, under ordinary circumstances, I must protest against, for this reason: first, because there has been before, and will necessarily be after extraction, irritation or inflammation in the gums, and alveola and around the natural teeth if there are any. At this time acid and irritating matter is secreted, and the dental substitute will, if inserted, instead of lessening the irritation, tend to increase it. Galvanic action will be produced and evil results follow.

Another reason against temporary substitutes is this: When the work is inserted, it must of course fit the gums over alveola before they are absorbed. The pressure of the plate keeps the alveola adapted to it during the absorption to a certain extent, leaving them in a very uneven and irregular shape. Again, as the alveola settles, the plate has an unequal pressure on some parts of the mouth, thereby keeping up a constant irritation.

And lastly, after temporary teeth have been worn for a time, they become badly adjusted, and often in this way habits of using the jaws are contracted which interferes very much with the good articulation of the permanent teeth.

I will here propose a few general directions by which we may avoid many of the evil results of which I have before spoken. In the first place, when a patient calls on me for advice and operations, I make a very careful and critical examination of the mouth and teeth, making up my mind as regards the pathological condition, and also what treatment I should propose to remedy the case. Then, if after informing my patient I have his approval, I carry out my plan in about the following system.

If there are any teeth which I propose to leave, I see that they are freed from all salivary calculus and foreign matter. Then I proceed to extract all decayed teeth and roots that cannot be saved by plugging. After the bleeding has subsided I remove all speculas of the alveola that would irritate the gums. As this part of the alveola would in time absorb, it is better to remove it at once by instruments and thereby hasten the process of nature. The gums are more comfortable and heal more rapidly and in better form. This I should invariably do in preparing for full sets. For unless they are removed, as the gums shrink away they project through the gums, keeping them sore for a long time.

The teeth all being extracted, if I find any other portion of the gums inflamed where the bleeding did not occur, I would bleed freely by the lance or leech, rubbing the gums briskly with the finger to facilitate the bleeding. After the gums have bled sufficiently to satisfy me, I rinse the mouth with a solution of tannin in water, which will arrest the flow of blood immediately.

This will constitute all of my operations during the first visit of my patient.

I next prepare a tonic and astringent wash composed of tinc. myrrh, cinchona, opium and orris root, to be used with water daily for some time, to keep up a tonic action in the mouth and keep down any traces of ulceration.

If there is any effect of salivation remaining, I also give them in addition a small solution of chloride of soda to counteract the effects of the mercury.

With the above, I recommend a constant use of tooth soap and powder to cleanse the mouth and teeth, with strict injunctions to be faithful in their use.

The above I always furnish my patients; for if I do not they are apt to neglect getting them, either from expense or carelessness. But if once they have them, they will generally use them according to directions.

I now dismiss my patients and direct them to call again in a few days.

At the next visit I remove any remaining traces of calculus and polish the teeth when it is needed, and if there is any inflammation existing I bleed the gums again, freely, and continue the lotions. As soon as all traces of inflammation are gone, I then proceed to plug the teeth, if they need it.

After this treatment, the remaining teeth are less liable to be sensitive than they would have been if plugged during the first operation, and more certain to be preserved by plugging. The directions to the patient should be strongly insisted upon with the reasons for so doing, otherwise they are liable to be neglected and the dentist will lose the honor of succeeding in his operations. For whatever the result may be, the patient will take no blame on himself; therefore, the dentist cannot be too strenuous in having his directions carried out.

The time necessary for the patient to wait before the substitute is inserted, will depend upon circumstances. For a temporary set at least one month, and for the permanent partial set not less than three, and full sets not less than eight months.

A very good guide in most cases is to wait until the furrow in the gum over the alveolar ridge is entirely obliterated before the substitute is inserted.

Patients are generally in great haste to get through, and to the dentist there is also something in the future to urge him to complete the work as soon as is possible. But the great object should be to perform such an operation as would be of the greatest service to the patient, and in the end the operator will not only be remunerated by pay for the work, but a satisfaction of knowing that his patient is truly benefited.

One more direction I would urge, which is this: that all artificial fixtures in the mouth should be taken out, and it and mouth thoroughly cleansed, at least three times per day. To this rule I would have no exceptions, for unless it is done, food and foreign substances accumulate around the natural and artificial teeth which produces a powerful chemical action which must do mischief sooner or later.

There are other causes of failure of artificial fixtures in the mouth; one of which is, that many patients prefer to get the cheapest operations, by which they secure not only badly adjusted fixtures, but what is worse, base metal. When such is the case, patients should not complain if the operation fails.

But unfortunately the patient is not the only one who suffers from bad operations; for the good operator comes in for his share of the abuse showered on the profession generally, for the bad operations of one of its members.

That artificial fixtures in the mouth are often the cause of great injury is, I do not for a moment doubt, but I am confident that the most of those results are from causes mentioned and may, in a majority of cases, be remedied by proper precautions.

The young members of the profession are more liable to fall into the errors mentioned; hence, instructors and writers cannot dwell too much on the importance of the primary operations. Otherwise, our operations will meet the name they sometimes get, of being fit only for "show work," not for utility. Our motto should be, first, *utility;* second, *beauty.* Then, and not until we work to our motto shall we be entitled to the praise we covet.

